# Analyzing non-technical skills in the sharp end of facilities/utilities operations in onshore and offshore O&G process plants

**DOI:** 10.1007/s10669-022-09884-w

**Published:** 2022-11-12

**Authors:** Josué França, Antônio Oliveira, Luciana Silva, Pär Karlsson

**Affiliations:** 1 Linnaeus University, Växjö, Sweden; 2 Federal University of Rio de Janeiro, Rio de Janeiro, Brazil; 3 Petrobras, Rio de Janeiro, Brazil

**Keywords:** Non-Technical Skills, Offshore Operations, Refinery Operations, Human Factors, System Safety, Resilience

## Abstract

It is on the sharp end of the O&G operations where the real work happens, but also where the highest risks and system demands are placed. Understanding the skills—technical and non-technical—necessary to perform efficiently and safely is not only needed to maintain the business and assets, but also to ensure the safety of lives and the environment. Accidents such as Piper Alpha (1988), P-36 (2001) and Deepwater Horizon (2010) highlight the importance of understanding the real role of the human element in these events, from the highest hierarchical levels to the sharp end, where the work as done takes place. This article presents a non-technical skills analysis focused on the sharp end of O&G operations, specifically in the facilities/utilities operations, onshore (refineries) and offshore (production platforms). The findings show the importance and presence of certain non-technical skills, as well as the need for improvement of others in the daily routine and in emergencies.

## Introduction

To perform any job, in any area of expertise, it is required specific technical skills, that involve the understanding of what is needed to be done, the necessary resources, the constrains and limitations. In addition, some non-technical skills are needed to perform a job within a specific socio-technical context. These non-technical skills, as teamwork, communication, leadership, decision-making and situation awareness, are the cognitive and social skills that complement the technical skills, enabling an efficient and safe operator performance in the work context (Flin et al., 2016). The ability to manage stress and cope with fatigue may also figure as non-technical skills, although there is some discussion, due its intrinsic nature, pleading to not include both as non-technical skills (Endsley, 2015; Fjeld et al., 2018). Contextualizing non-technical skills in the Oil & Gas (O&G) domain, it is noticed that they have a relevant and inseparable role, being associated with the productive and safe development of work activities in onshore and offshore environments (Flin et al., 2016).

On the other hand, their absence or omission have been identified as one of the factors that compose the causal structure of incidents and accidents (Sneddon et al., 2006). Therefore, the understanding of these non-technical skills characterized in this domain, allows a wide comprehension of how it can contribute for a safe and productive work, ultimately helping to prevent accidents. During the period 1,982,010, studies have been performed on more than 600 well-documented system failures in offshore platforms, involving a wide variety of types of engineered systems, to understand the roles of the various components that comprised the systems during the life-cycle phases that led to the failure (Bea, 2011). The analyses involving organizational, social, cognitive and behavioural elements, have not been developed properly, being mistakenly directed to individual failures and labelled as human error (Vaughan, 2016). Not only in O&G NTS are relevant, also in healthcare area there is a growing recognition that the attainment of expert proficiency requires persistent active learning, focusing training to gain both (and integrated) technical skill and NTS (Raison et al., 2018).

**Fig. 1:**
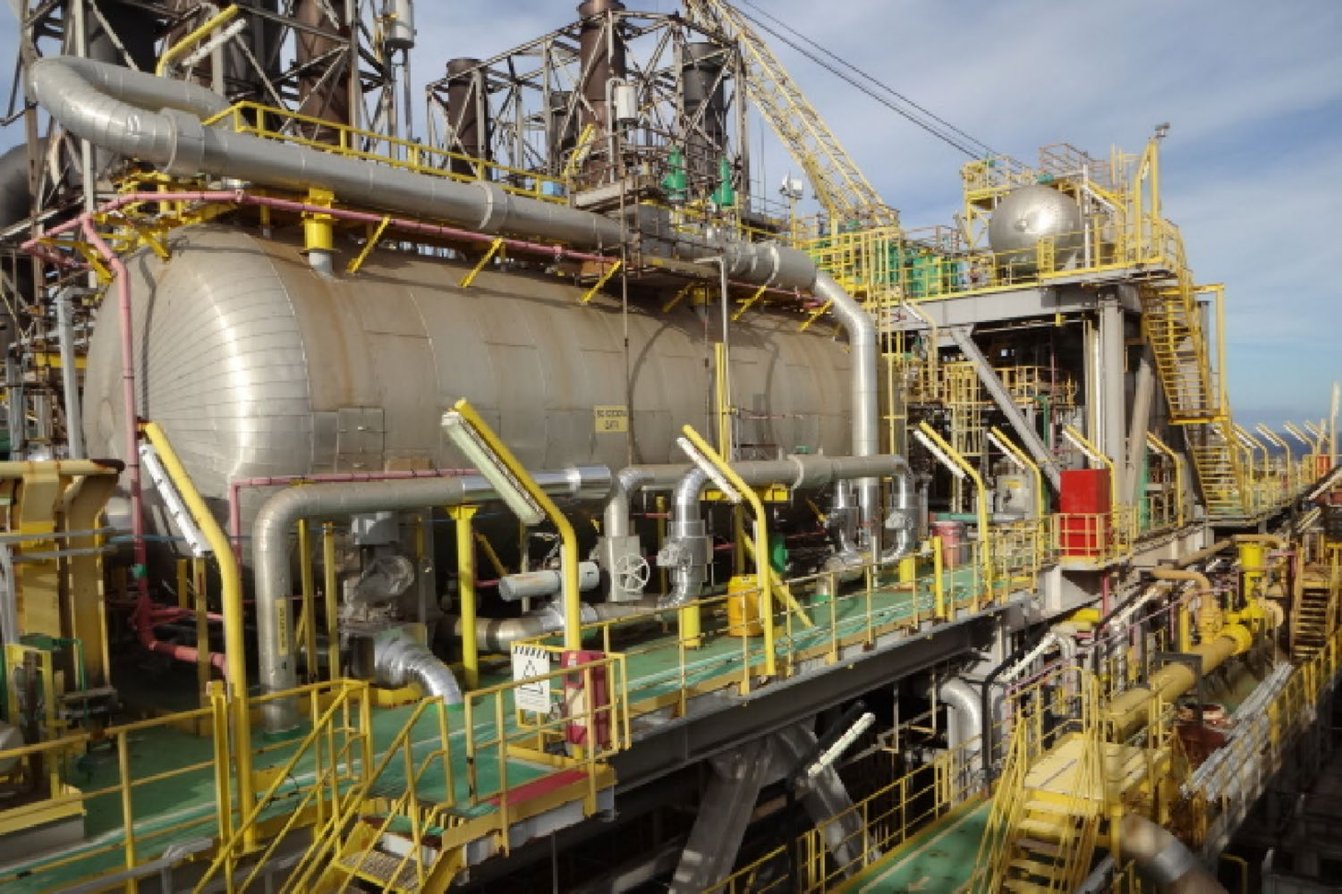
Process plant of the power generation unit in a FPSO. Source: Authors (2021)

Thus, there is still a need for a systemic and integrated understanding of how the interactions occur in workplaces, in order to adequately comprehend the human factors in all their dimensions—technological, environmental, organizational and individual—and determine how these interactions happen, in which levels and how contributes for daily bases work and accidents. In view of this need and based on the studies of human factors and non-technical skills developed in the O&G area (Gordon, 1998; Sneddon et al., 2006; Skalle et al., 2014; Flin et al., 2016; Strand & Lundteigen, 2016; França & Hollnagel, 2020), an analysis of non-technical skills is presented in this article, in order to understand the cognitive and social elements that are naturally present in work environments. This is important as the Oil & Gas domain is characterized as complex sociotechnical systems, with a high degree of technology, intense human interactions, critical process variables, and a certain degree of uncertainty. Figure 1 depicts the process plant of the power generation unit in a FPSO, a Floating, Production, Storage and Offloading platform, where the utilities operators perform their activities daily basis.

Seeking to understand these interactions that occur in such complex sociotechnical workplaces, this research explores and discuss the role of the non-technical skills in the sharp end of facilities/utilities operations in onshore and offshore O&G process plants. Therefore, some questions support the hypothesis of this research:

1. Observing the routine work of facilities/utilities operators, what are the most present non-technical skills?
2. From the operators’ own perspective, what are most relevant non-technical skills to perform their work?
3. What are the differences/coincidences in non-technical skills relevance on routine and emergency situations?

## Characteristics and evolution of workplaces in the O&G domain

The evolution of technology in the engineering systems of O&G industrial plants—platform, refineries and terminals—is remarkable. Since the first oil well drilled in 1859 (Yergin, 2008), the system, machinery, process variables and procedures have evolved significantly. However, there is an element that remains the same in the terms of its evolution but is the driving force of all that: the workers that project, build, operates, maintain and uninstall all those systems. In this sense, to understand of how the current offshore complex sociotechnical systems works, analysing their limitations and capabilities through their own technical evolution, it is necessary to develop a comprehension not focused on the worker, looking for his flaws, but as from the workers, understanding their limitations, capabilities and their natural resilience that keeps them productive in work routines, and prepared for emergencies and contingencies.

### Non-technical skills in the O&G domain

Non-technical skills (NTS) are defined as the cognitive and social skills that complement technical skills and contribute to safe and efficient task performance (Flin et al., 2016). In this article, five of these are addressed, due their importance in the context of work: communication, leadership, teamwork, decision-making and situation awareness. Analysing the work itself from this perspective, to perform a task, it is necessary to have both NTS and technical skills, being this set defined as a professional skill, which is a particular way to perform a job (Wachs et al., 2012). The evolution of the studies on NTS started in the civil aviation, characterizing and being part of the CRM (Crew Resource Management) programs, but currently is not limited to this domain, being perceived in healthcare and energy business (Wachs et al., 2012). When contextualized in the O&G area, onshore and offshore, where both are high complex, high risky and high skilled in workplaces (Robb & Miller, 2012; França et al., 2019), the understanding of this set seems reasonable and adequate for comprehend the natural resilience present in daily basis operations, as well in emergency situations.

In contrast to technical skills, NTS do not have a specific domain to be placed, being observed in several different work activities and environment. Once it is a phenomenon that emerges from social and cognitive interactions, its presence in complex sociotechnical workplaces is commonly perceived. Traditionally, NTS have been identified, developed, and applied in the aviation domain (Wiener et al., 2010). However, as NTS are recognized in work activities within the context of a complex sociotechnical system, there are studies in the maintenance (Irwin et al., 2016), healthcare (McCulloch et al., 2009), energy production (Wachs et al., 2012), IT (Danaher et al., 2019) and O&G areas, such as the one presented in this study. The NTS presented by (Flin et al., 2016) was adopted in this research because the work activities under analysis are in the O&G industry, precisely in the facilities (platforms) and utilities (refineries) operations. Moreover, several scientific studies comprise the same NTS presented by (Flin et al., 2016) as appropriate and applied to the O&G industry segments (Moffat & Crichton, 2015; Crichton, 2017; Thomas, 2018) and (França et al., 2020). Reinforcing these NTS on offshore activities, some studies presenting its importance on the maritime domain, which not only includes O&G vessels, but also shipping, passenger, supply and others (IMO, 2010). These studies converge on the strategic importance of communication, leadership, teamwork, decision-making and situation awareness for safety in the onboard activities at the sea (Fjeld et al., 2018; Tavacioglu & Gokmen, 2020) and (Praetorius et al., 2021). Some of these studies, despite being focused on a certain domain, have developed an argumentative nexus between human competences and the resilience capabilities of the system where they are inserted (Wachs et al., 2012; Praetorius et al., 2021). Therefore, it is possible to notice an intrinsic relationship between the resilience of a work system and the ability of workers to adapt to complex demands, reflected in their skills, both technical and non-technical.

Resilience can be understood as the intrinsic ability of a system to adjust its functioning prior to, during, or following changes and disturbances, so that it can sustain required functionality under both expected and unexpected conditions (Hollnagel et al., 2011), being object of study of Resilience Engineering. Once this engineering aims to develop the safety of complex systems, such as onshore and offshore O&G facilities, by measuring and improving its resilience, there is an inherent relationship between this discipline and the analysis of skills, both technical and non-technical, in complex sociotechnical systems. Indeed, in O&G domain, as well as in the maritime (Grech et al., 2008), the resilience can be understood as the ability to sustain required functioning and achieve system goals under a variety of operational conditions (Praetorius et al., 2015). Also, from the Resilience Engineering perspective, the understanding of NTS does not relay on only in the individual worker’s skills, but also a supportive working environment is also necessary, analysing the whole sociotechnical system (Wachs et al., 2012). Taking the teamwork as an example, it is possible to notice that individual contributions build the collaborative work, which is the intrinsic characteristic of teamworking (Flin et al., 2016). And, at the same time, workers shape their individual behaviour to be part of the team, characterizing this duality between individual and collective.

Analysing these concepts within the O&G area, it is possible to notice that both offshore and onshore operations plants, such as oil rigs and refineries, presents the characteristics of high reliability organisations, which are a reluctance to simplify, a preoccupation with failure, sensitivity to operations, a respect for expertise, and resilience (Thorogood & Crichton, 2014). Indeed, most of the workplaces of the O&G area, once involves high technology, system automation, process control, multiples production stages and intense human interaction, are characterized as a complex sociotechnical system. The work itself is usually highly specialized, through many tools and information systems, dealing with high process parameters, being uniquely dynamic and constantly changing and adapting (Reiman & Oedewald, 2007). In such work and workplaces, the awareness of operators within environment and equipment, combined with recognition of signs that allows prediction of the consequence, can not only promote a desired performance, but also avoid near misses and unwanted consequences (Irwin et al., 2016). The human element, therefore, acts as an active and dynamic barrier for the entire process, having its skills, technical and non-technical, as necessary tools to read, interpret, recognize, and act in face of system demands and alterations.

In these work environments, and in their daily activities, O&G operators, thus, need to demonstrate high levels of not only technical performance but also non-technical, like team attitudes and behaviours, to operate safely and adaptively to achieve their goals (Crichton, 2017). And to achieve this performance, it is needed a combined development of training and understanding of non-technical skills. According to (Moffat & Crichton, 2015), the O&G industry recently acknowledged the importance of NTS training for safety operations, for daily operations and emergency responses, implementing an initial course for offshore control room operator competence assessments and emergency response training. This initiative was the prototype CRM course for offshore platform, which one was commissioned by IOGP (International Association of Oil and Gas Producers) Report 501, establishing the basis of CRM practices for well operations teams (IOGP, 2020). This report recognises the importance of NTS training to operational safety in the area of well operations, embedding discipline-relevant skills and attitudes not only for the training itself, but also for good operational practises (Moffat & Crichton, 2015). Indeed, for (Thorogood & Crichton, 2014), NTS can be effective observed, coached and assessed on a routine basis in the workplace, enabling the operators to be prepared for the unusual situations that may arise, wielded by their own technical and non-technical skills.

### Systems resilience in the O&G domain

The origin of resilience concepts is rooted in ecology, associated to the ability of an ecological system to achieve, after an adaptative response, a stable state (Hollnagel et al., 2006). This concept highlights that the balance achieved through adaptation over time, in a dynamic and changing environment, requires constant changes in the functioning of a system. Based on these concepts, within the resilience engineering framework, resilience has been defined as the ability to sustain required functioning and attain to operational goals under a variety of operating conditions (Hollnagel et al., 2011). Therefore, resilience engineering grounds on the understanding of the systems performance in changing operating environments with an emphasis on how safe performance is achieved. Based on the premise that work environments in the O&G domain are true complex sociotechnical systems (Righi & Saurin, 2015), its functioning is the result of complex interactions between people, systems, equipment and processes, immersed in a particular organizational culture, being this relationship in dissociable. In this way, the relationship of NTS with resilience forms the adaptive dynamics of the system, with people, workers, the enabling element of this adaptability. It highlights how work systems successfully adapt their functioning to the complex demands of sociotechnical systems, such as those that form the workplaces of the utilities (offshore) and facilities (onshore) areas.

It can be seen, then, that there is an interdependence between the complexity of the sociotechnical systems and the high training NTS capacities of the operators who will work on them and seems that more the level of complexity and technology increases, the more human interaction is required. Dynamic situations in this system requires that teams, particularly in the sharp end of O&G operations, are trained to react in an effective and timely manner, monitoring simultaneously system parameters, performing regular routine procedures, and quickly responding to non-routine critical situations (Crichton, 2017). In this sense, the accurate perception and comprehension of environmental information is linked to the process of decision-making and task-based actions, which is, in fact, the assertiveness and adaptability of the operators (Irwin et al., 2016). Once that one of the most visible manifestations of resilience in the sharp end operations is the individual adaptabilities of the workers, the study of the NTS mechanisms arises as a way of comprehending these interactions and behaviours (Wachs et al., 2012). Therefore, seeking to understanding this mechanism and the importance of the human element in a sociotechnical system, the analysis proposed by this research is focused on the daily basis activities of the sharp end, both in onshore and offshore operations, considering the non-technical skills naturally present herein.

## O&G operations in the sharp end

The activities under research are the offshore and onshore operations, performed by operators who are working in the sharp end, at the last stage of the O&G production chain. For onboard operation, in the offshore platforms, the operations are performed mainly by three different operators, which are:

- Production operator: responsible for the process. responsible for all the production, processing, storage and transferring processes of hydrocarbons produced by oil wells, which are natural gas and oil, the latter with about 24 API grade (Campos Basin average). It is a fact that all areas of a FPSO are essential for its functioning, however, historically speaking, and due to its complexity and potential for accidents, the production area is the most critical of an offshore oil platform (Figueiredo, 2016).
- Stability operator: responsible for the stability, buoyancy and (dynamic) positioning of the FPSO. In most of the cases, the FPSO is an old large oil tanker converted to a FPSO production platform. So, these operators are more seafarers than operators itself, once their job is more related to a ship than an offshore platform. In fact, most of the training and procedures regarding this function is regulated by IMO (International Maritime Organization) and Brazilian Navy, once these platforms are in Brazilian territorial waters.
- Facilities operator: responsible for the infra-structure resources for production and other areas, being responsible for HVAC system, water system (hot, cold, salt and process water), compressed air and electrical supply.

In the other hand, for onshore operation, in refineries, the operations are performed mainly by three different operators, which are:

- Production operator: there is a very wide range of operators, different for each process unit, having up to ten more specialties in a plant, from the atmospheric distillation to the special treatment of the petroleum derivates.
- Transfer and storage operator: responsible for the pipelines, valves, tanks, spheres and other equipment which connects and storage all the fluids of the refineries, whether petroleum and its derivatives, as well as other products needed by process units.
- Utilities operator: responsible for the infra-structure resources for production and other areas, being responsible for vapour, compressed air and electrical supply of the entire process plant, as well as supplementary system of the refinery.

## Materials and methods

Based on the 32-item checklist proposed by Tong et al (2007), the methodological approach adopted in this research will be detailed and explained. This checklist, denominated COREQ, aims to promote complete and transparent reporting among researchers and indirectly improve the rigor, comprehensiveness and credibility of interview and focus-group studies (Tong et al., 2007). Despite being primarily designed for ​​healthcare area, it has been used in other domains (Finstad et al., 2021), as well translated for other languages for national application (Souza et al., 2021). These 32-items were delineated for qualitative studies and precludes generic criteria that are applicable to all types of research reports, covering the necessary components of a research methodology and formed by three different domains (Tong et al., 2007):

1. Research team and reflexivity: personal characteristics (questions 1–5) and relationship with participants (questions 6–8).
2. Study design: theoretical framework (question 9), participant selection (questions 10–13), setting (questions 14 a 16) and data collection (questions 17–23).
3. Analysis and findings: data analysis (questions 24–28) and reporting (questions 29–32).

### Research team and reflexivity

Two researchers were dedicated to the observations, questionnaires and interviews phases, being assisted by the others to consolidate all the data and perform the final review. Both have higher academic background in social sciences, experience in conducting research and lecturing scientific disciplines. There was no previous relationship established prior to study commencement between the research team and the workers. The observations and questionaries phases were developed without purposeful interaction with operators, while in the interviews there were interactions between participants and interviewers. Before the start of each interview, the researchers introduced themselves to the operators and explained the objectives of the study. The participants of this study are the Utilities operators, working in refineries, and the Facilities operators, worming in offshore platforms.

### Study design

**Fig. 2:**
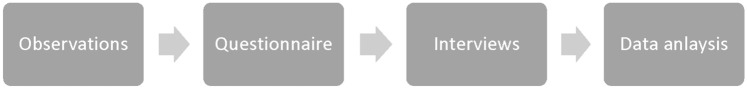
Representation of the methodological approach. Source: Authors (2020)

The methodological approach designed for this study is consisted of four consecutive steps (Fig. 2).

#### Offshore and onshore observations

The study started by naturalistic observations onshore and onboard. This type was chosen because allows to study behaviours and attitudes of participants in the field, in the sharp end (Patton, 2002). In this research, the sharp end is the onshore operations, in refineries, observing the work of Utilities operators; and offshore operations, in production platforms, observing the Facilities operators. These observations were done without interfering in the work of the operators, but those who asked questions about the survey were answered. Through direct observations in the field, it is possible to understand and capture the context of work and interactions, giving a holistic perspective (Patton, 2002) even when the observations are open and passive, with no planned interaction from researchers. Each observation had a duration between one to two hours, considering different times of work shift: morning, afternoon, and night. The observations were recorded by notes, and only few photos were allowed, being part of this study.

The onboard observations were in one SS (semi-submersible) platform and in two different FPSO, all of them located in the Campos Basin area, in Brazil offshore O&G province. The observations in the refineries were done in four different refineries in Brazil, one located at Rio de Janeiro, two located in São Paulo e one located in Minas Gerais, in the southeast region of the country. The observations were done without interaction with the workers, aiming to harvest data and insights to develop a basis for the qualitative ethnographic study of non-technical skills (Guest et al., 2012). The observations were performed before March 2020, when officially decreed by WHO the COVID-19 pandemic, and Brazil started the national restrictions and protective measures to prevent the virus spread in the territory.

#### Questionnaire

Once the field observations settle the basis for the research, the second step was a one-page questionnaire, printed and sent by e-mail, to be answered by operators who were working at the sharp end of offshore and onshore O&G operations. The printed questionnaires were delivered by mail and in person, being this last one under strict protection protocols. In all, 124 questionnaires were sent by e-mail, in PDF format, for 62 Utilities operators (onshore) and 62 Facilities operators (offshore), being equally divided into offshore and onshore units. The one-page questionnaire was chosen because it is giving a more user-friendly approach for gathering data (Coyle et al., 2016), and promotes an objective and effective communication to all involved in the project, participants and researchers (Campbell & Campbell, 2013).

The questionnaire consisted of seven items designed to gather data on the perceived relevance of NTS for the sharp end operations. The questionnaire used a five-point Likert-scale to address this data. Although the one-page questionnaire was adopted, in fact, the two sides of a single sheet were used as the questionnaire. On the front side, using the Likert-scale itself, seven questions were posed, based on the five NTS previously presented. The questions, translated from Portuguese to English, and focused on the daily work routine are:

1. In your opinion, what is the relevance of **Situational Awareness** in your daily work routine?
2. In your opinion, what is the relevance of **Decision-Making** in your daily work routine?
3. In your opinion, what is the relevance of **Leadership** in your daily work routine?
4. In your opinion, what is the relevance of **Communication** in your daily work routine?
5. In your opinion, what is the relevance of **Teamwork** in your daily work routine?
6. Considering your real work, performed in your daily routine, which **NTS is most present**?
7. Considering your real work, performed in your daily routine, which **NTS is most neglected**?

And the answers, based on a Likert scale itself, translated from Portuguese to English, for the 1–5 questions, and then for 6–7 questions, are:

1–5 questions:

**Table:** 

Extremely important	Very important	Reasonably important	Little important	Not important

6–7 questions:

**Table:** 

Situational Awareness	Decision-Making	Leadership	Communication	Teamwork

On the second page of the questionnaire, questions included figures and emoji’s as response alternatives instead of the traditional Likert-scale. The aim was to analyse how the response would be between the traditional written scale, with an eminently visual scale, once that human interactions with system are stimulated when visual elements are involved (Ernst & Banks, 2002). Indeed, in terms of cognition, (Damásio, 2005) presents those social interactions with visual stimulus trigger brain circuits related to emotions and memory, generating intense brain activities and consolidating memory. Based on that, eight questions were proposed, considering the five NTS previously presented. The questions, translated from Portuguese to English, and focused on the emergency and contingency situations are:

1. In your opinion, what is the relevance of **Situational Awareness** in emergency and contingency situations?
2. In your opinion, what is the relevance of **Decision-Making** in emergency and contingency situations?
3. In your opinion, what is the relevance of **Leadership** in emergency and contingency situations?
4. In your opinion, what is the relevance of **Communication** in emergency and contingency situations?
5. In your opinion, what is the relevance of **Teamwork** in emergency and contingency situations?
6. Considering the emergency and contingency situations of your work, which **NTS is most present**?
7. Considering the emergency and contingency situations of your work, which **NTS is most neglected**?
8. Considering **only the graphic presentation and methodological structure**, disregarding the questions themselves, which of the two sets of questions did you feel most comfortable answering?

And the answers, structured as Likert-scale, but with figures and emojis, translated from Portuguese to English, for the 1 to 5 questions, 6 to 7 questions and 8 question are:

1–5 questions:
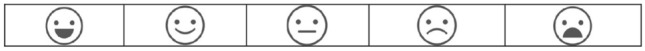


6–7 questions:
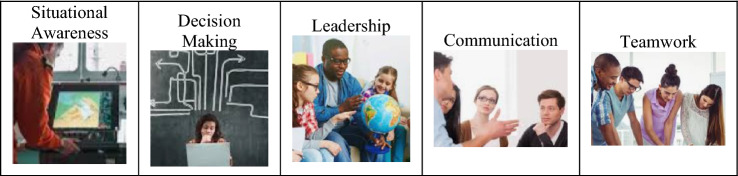


8 question:

**Table:** 

NTS, 1st Part Work routine (tables and phrases)	NTS, 2nd Part Emergency and contingency situations (emoji and figure thumbnail)

The eighth question was proposed to perceive and analyse the willingness to answer, comparing written information with visual information, considering the visual stimulus in the social interactions that activates some brain functions (Damásio, 2005). Also, to have an open channel of communication with the subjects and have insights for the online non-structured interviews, a special space was reserved in the final part of the back side, just after the eighth question:

**This space is yours! Write here your opinion/comment that you consider relevant about NTS:** 
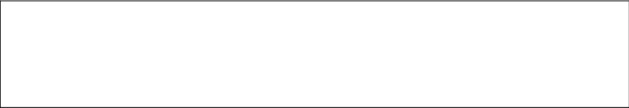


Your contribution is very important to this study. THANK YOU VERY MUCH!

Aiming to recognize and be grateful to everyone, a final message of thanks and disclosure of the importance of participation in the study was highlighted, right after the blank space for writing.

#### Online non-structured interviews

The online interviews were performed by computer, using the meeting software Zoom® or Microsoft Teams®. Also, some interviews were performed by WhatsApp® calls, using the video function to seize the reactions and expressions of the operators. The approach applied in this part of the research to collect data were the standardized open-ended interview, where a set of questions is meticulously worded and arranged with the intention of taking each respondent through the same sequence of questioning (Patton, 2002). The three questions developed to structure this stage of the research were:

- Please tell me about your work, your routine and emergency situations (if it has already occurred);
- Please, in your opinion, what is the role of these NTS in your work, both in routine and emergency situations?
- Please, is there anything you would say, add or criticize? It is an open channel, feel free to talk.

Sixteen interviews were conducted, eight with offshore operators, and eight with onshore operators, to establish equity, and none of that were recorded. Several notes were taken from the interviews. The interviews were conducted by two researchers, following the sequence of questions presented. After each question, the researcher waited for each of the interviewees to finish their answer, being noticed different response times among the workers. The interviews were not recorded, as the worker’s companies did not allow this protocol. Thus, the researchers took notes of the answers, which were compiled and analysed further on.

The answers from the first question revealed that the work of operators in facilities and utilities is complex, compelling them to take all possible resources to deal with routine activities, as well as emergencies. From these skills, technical and non-technical, emerges a unique ability to know (technical skills) and perceive (non-technical skills) the functioning of the entire process plant. This clearly demonstrates that human action is a dynamic barrier to process safety, adapting to the sui generis demands that emerge from a complex sociotechnical system. In fact, one of the utilities operators reported that “feeling” what is happening in the external area is essential for his work: “—And then, that day… I felt this strange noise… as if it were water dripping from the faucet… That bothered me… I had never heard that before! So, I kept looking, looking and then I found the turbines all frozen, with gas leaking everywhere! I immediately call the panel by the radio, and we were able to stop everything, without any flash, and without anyone getting hurt… That day was punk and I really got scared!”. This testimony was translated from Portuguese.

The second question answers demonstrated that part of the interviewees really knew what NTS were, part had an idea, but without details, while a small group declared not knowing about NTS. Despite this, all were unanimous in stating that NTS are essential for their work, citing real examples related to their performance. One of the workers who did not know the NTS until then, made a statement of how much it is present in his routine: “—Well, you know, right, we have to do a lot of sampling… That day, I went to do a pipeline sampling, in the sampler that is on the main deck. As there was a lot of corrosion, the sampler broke in my hand, and I had to decide what to do in seconds, otherwise it would have diesel all over the platform and blow everything up! During my walk routine, I saw some scaffolding tubes on the floor, and then I took one of them to support the valve, before it broke for good. Only then I call the maintenance guys by radio, saying it was top urgent and I was already completely full of diesel. The maintenance supervisor, who is also a brigade member, heard everything on the panel and ran along with the team, just to keep throwing water on me. In the end, in less than 20 min, everything was under control and a provisional plate was installed in the pipeline. I went to take a shower, because although the supervisor had soaked me in water, I really reeked of diesel. Did you see? All these NTS that you mentioned are there! We had the communication, the supervisor’s leadership, the teamwork of the maintenance guys, my decision-making and my situational awareness of using whatever were there. We are like that, we solve everything, every day, using everything we have.”. This testimony was also translated from Portuguese.

In the third question, there were several criticisms, from the simplest, involving elements related to the living areas, to relevant structural issues, such as the acquisition of inappropriate materials, with inferior quality and quantity, requiring constant adaptability from operation. Despite the utilities operators being at the refineries and, in theory, not suffering from the logistical problems of the arrival of materials, as in the offshore platform, the same inadequacy was reported. Most reports mention that the most critical organizational factor is the outsourcing process, where the provision of services, or supply of materials, can be contracted for the best technique or the lowest price, being this one predominantly adopted. Consequently, there are several failures of this origin, as reported by one of the facilities operators: “—Every time we have to do a manoeuvre in the generator room, we know we have to take a certified flashlight, because all the time the lamps there, which are explosion-proof, have a burned bulb and there is no way to see anything! And every time they change this bulb, every time it fails! The maintenance people have already said that this Ex supplier sucks, that they don’t have a single Ex equipment that works fine… But as long as our guys onshore keep buying at the lowest price… We will keep having this type of inadequate equipment installed onboard.”. This testimony was also translated from Portuguese.

Within these universes of operators and considering the differences between the offshore and onshore areas, it was determined to develop research that could focus on a certain niche, but at the same time consider an analysis that is also comprehensive. Thus, the questionnaire was sent to all operators who were willing to participate, but the observations and interviews were only focused on those operators who carry a certain similarity: utilities (offshore) and facilities (onshore). Despite all the observations were carried out before the pandemic situation decreed by WHO, all the interviews were done online, once this public health status was already established. Some limitations were noticed, especially related to online connection resources. When these resources were scarce, failures and delays affected video and voice reproductions. Some of these were fixed by a new call, but few of them were needed to be rescheduled, for another time or day, using the same online platform, or changing for another.

### Analysis and findings

The data consolidation showed that 28, 22% of the questionnaires were responded. From the ones which were answered, seeking to ensure data reliability and consistency (Saris & Gallhofer, 2014), the questionnaires that were strikethrough, or had double answers in one or more question, or had no answer in one or more questions, or was erroneous answered by a different operator of the mapped ones, were completely disregarded. In Table 1 is possible to see the results of the answered questionnaires.

**Table 1:** Results of the answered questionnaires

		Sent	Received	Disregarded	Considered
Offshore	FPSO 1	20	11	3	8
	FPSO 2	22	5	0	5
	SS	20	3	1	2
Onshore	Refinery 1	16	6	1	5
	Refinery 2	15	3	1	2
	Refinery 3	15	8	2	6
	Refinery 4	16	9	2	7

Analysing the answering percentage of 28, 22%, where 35 questionnaires of 124 were fully considered the study, the data collection may seem little for debugging. However, when analysing the absolute number of 35 questionnaires, it is noticed that there is a possibility of coherent and consistent analysis. When there is sufficient data to reflect the proposed research, supported by field observations, there is the material necessary for scientific research (Saris & Gallhofer, 2014).

One of the reasons of the low response falls on the workload of the operators, preliminary observed in the field studies and several times reported by interviewed. Most of the sharp end operators are working in their premises, although there are few of them working remotely from home, what is physically felt in the operational area, with consequent work overload. As the pandemic continues to threaten health, is perceived a prolonged period of this way of working—few from home and most in the sharp end—which gives different proportions and loads of work.

## Results and discussion

The consolidation of the 35 questionnaires considered in this study brings some relevant information regarding the five NTS under study. Table 2 presents the NTS importance in daily routine basis, from 1 to 5 question.

**Table 2:** Results of the NTS importance in daily routine

	Extremely important	Very important	Reasonably important	Less important	Not important
S. Awareness	15	18	2	0	0
D. Making	17	17	0	1	0
Leadership	8	15	9	1	2
Communication	28	7	0	0	0
Teamwork	26	9	0	0	0

Analysing all these answers, it is noticed that communication is the most relevant NTS, and indeed is present, because is really needed for daily routine operations. In the sharp end, where the real work happens intensely, spoken communication is both social and functional. At a social level, build relationships and bound trust, whereas is the instrument to help a team to carry out daily activities (Flin et al., 2016). In this sense, communication can be compared to a cement, that aggregates all the NTS together, as much as technical skills and individual characteristics. The worker’s skills, experience and perceptions flow through the work activities carried out by the communication, enabling productive and safety outcomes. The observation onboard and in the refineries confirm this importance, covering all forms of communication: written, non-verbal, by radio, by the system (e-mail or chat) and the spoken language itself. Other studies in O&G area have shown that communication plays a fundamental role for the safe execution of the activities (Sneddon et al., 2006; Abimbola et al., 2014; França et al., 2019). In the online interviews, the communication was pointed as fundamental for all activities that involve risks, being an essential bridge to build the social connections between people and teams.

Another NTS that arises as relevant is the teamwork, which is intrinsic related to communication, once it is this last one that allows the teams to connect themselves, using verbal and non-verbal channels. Teamwork is a key issue for most work areas, however, is especially important in complex sociotechnical workplaces, such as FPSO and refineries. Considering the routine activities, the absence or abjection of teamworking can mislead to potential harmful outcomes. The observation onboard and in the refineries confirmed the relevance of the teamworking, being also highlighted by online interviews as essential for a good and safe work. The questions 6 and 7, regarding the most present and the most neglected in the daily routine, consolidated in the Table 3, confirmed the importance of the duality between communication and teamwork, but also pointed out that situation awareness needs improvement.

**Table 3:** Results of the most present and neglected NTS in daily routine

	Situation awareness	Decision-making	Leadership	Communication	Teamwork
Present	6	5	5	8	11
Neglected	10	7	9	6	3

**Fig. 3:**
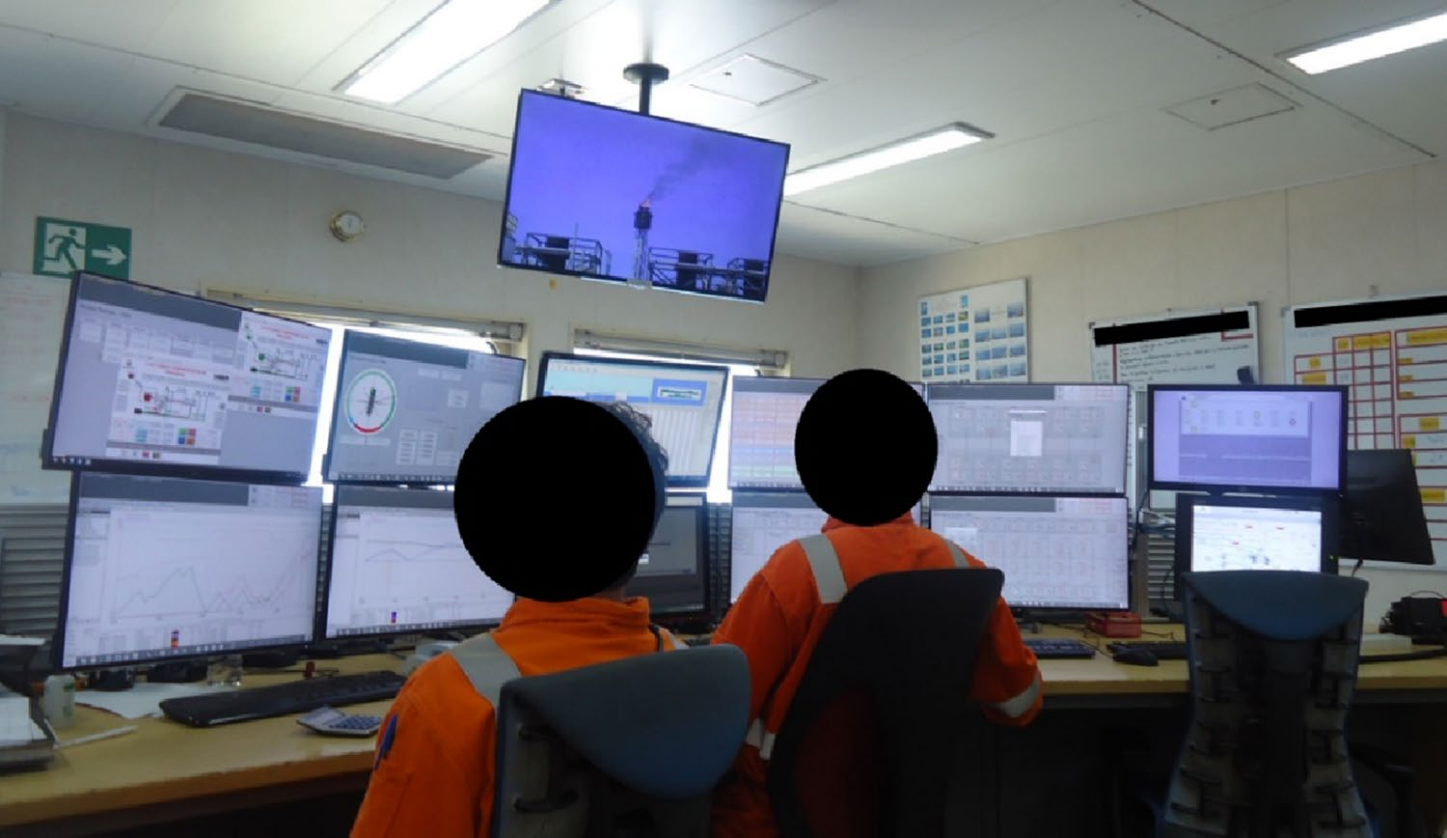
Control room activities for utilities operations of a FPSO. Source: Authors (2021)

Situation awareness is the way of perceiving of what is going on in the workplace. It is the perception of the elements in the surroundings within a notion of time and space, the understanding of their meanings and the projection of their status in the near future (Flin et al., 2016). Even when individually is present, only when it reaches collective levels, features related to safety are observed. Indeed, situation awareness can be defined as an overall understanding of the current situation, based on the extraction of relevant information from the environment and prior experiences from workers (Stanton et al., 2020). Analysing this from the micro to the macro level, the individual risk perception is a cognitive structure for the development of the situation awareness, which emerges individually, but generates results collectively. Onboard the FPSO, this NTS is highly present in the control room, where the number of screens, alarm flooding, radio/phone calls and other systems interactions demands a high level of awareness of the operators, as presented by Fig. 3. It is a routine activity, which is done in a 24-h work-shifts, once the utilities operations are continuous.

One of consequences of increasing the level of technology in the O&G process, and consequently, the complexity of the interactions in sociotechnical system like FPSO and refineries, is the decreasing of situation awareness (Grech et al., 2008). Furthermore, hierarchical pressure, inadequate workload, organizational culture and the other NTS have critical influence in the levels of the situation awareness. Complementary, from the second page of the questionnaire, in Table 4 presents the relevant NTS in situations of emergency and contingency, from 1 to 5 question of the 2nd part of the questionnaire.

**Table 4:** Results of the NTS importance in emergency and contingency

	Extremely important	Very important	Reasonably important	Less important	Not important
S. Awareness	18	12	5	0	0
D. Making	22	9	4	0	0
Leadership	13	16	6	0	0
Communication	23	10	2	0	0
Teamwork	22	11	2	0	0

There is, therefore, an opportunity to improve this competence, developing it mainly in a practical context. In fact, situational awareness is the skill that structures the situational context of work activities, where people’s performance is grounded and, consequently, the application and development of other NTS (Salas & Dietz, 2011). Thus, one of the ways to foster this NTS is justly the recognition of the elements of a work environment, within a practical situational and temporal context. One of the techniques that can support this recognition is the briefing and debriefing, as both precisely allow this recognition through the workers themselves, in the daily practice of their activities (França et al., 2021). The promotion of situational awareness through briefing and debriefing tools, in addition to the integrated development of NTS, also develops the resilience of the sociotechnical system that forms the workplaces (Praetorius et al., 2021). Therefore, aiming to encourage, develop and apply situational awareness in the scenarios highlighted by this study, it is proposed to implement briefing and debriefing tools in the activities performed in the utilities (offshore) and facilities (onshore) areas.

As noticed already for the routine activities, communication also stands out as the most relevant NTS for situations of emergency and contingency. And specially in emergency situations, when multimodal channels are applied—radio, gestures, verbal and iron-mouth broadcast—the communication can be inefficient or misunderstood (Grech et al., 2008). The setup of clear and objective communication channels is the best way to deal with emergency situations, in O&G industry, and in any other complex industries. Accidents such as Piper Alpha (1988), AF Flight 447 (2009), Deepwater Horizon (2010) and Fukushima (2011) showed that as harmful as an accident is, if there are communication failures during the critical moments of the emergency, the consequences can become worse and affect generations. In this sense, training focused on NTS, such as CRM (Crew Resource Management) and BRM (Bridge Resource Management) provides a way to understand how the dynamics of communication during critical situation happens, developing this particular NTS integrated with technical skills and worker’s expertise.

Two other NTS arises as relevant for emergency and contingency, having the same score from the questionnaire: Teamwork and Decision-Making. Teamwork, as previously noted, is important for daily routines activities, and remains important for critical situations as well. Decision-making can be defined as the mechanism of reaching a judgement of choosing one, or several options, to achieve the needs of a given situation (Flin et al., 2016). It is a dynamic response for the system, and depending on other NTS, such as situation awareness and communication, once the decision is inserted in a context and demands for actions, generating consequences. The decision-making is not an easy process at all, choosing several functional areas of the brain, its hormones, circuitry and memory, and in most cases, acting in milliseconds (Damásio, 2005). The questions 6 and 7, regarding the most present and the most neglected in daily routine, consolidated in Table 5, confirmed the importance of the duality between teamworking and decision-making, but also pointed out that situation awareness and communication has a need of improvement.

**Table 5:** Results of the most present and neglected NTS in emergency and contingency situations

	NTS in emergency and contingency				
	Situation awareness	Decision making	Leadership	Communication	Teamwork
Present	5	8	8	6	8
Neglected	11	6	4	9	5

Once more, as evidenced previously in the analysis of daily routines, situational awareness appears neglected, which makes clear an alert for activities in the sharp end of the O&G operations, for moments when the process is running smoothly, and also when is out of control. It comes up as an important sign, showing that the response is coming and acting, but may not be at the level of the necessities, once can have a flawed assessment of the situation. An adequate assessment of the situation, done by a communicative team, can generate appropriate decisions, leading for an appropriate response. The operator’s non-technical skills, individual characteristics and technical skills, integrated, and present in their actions in daily routine or in emergency situations, brings up the system resilience, providing desirable outcomes, and ultimately, saving lives, as the emergency river landing of US Airways Flight 1549 (NTSB, 2010).

**Fig. 4:**
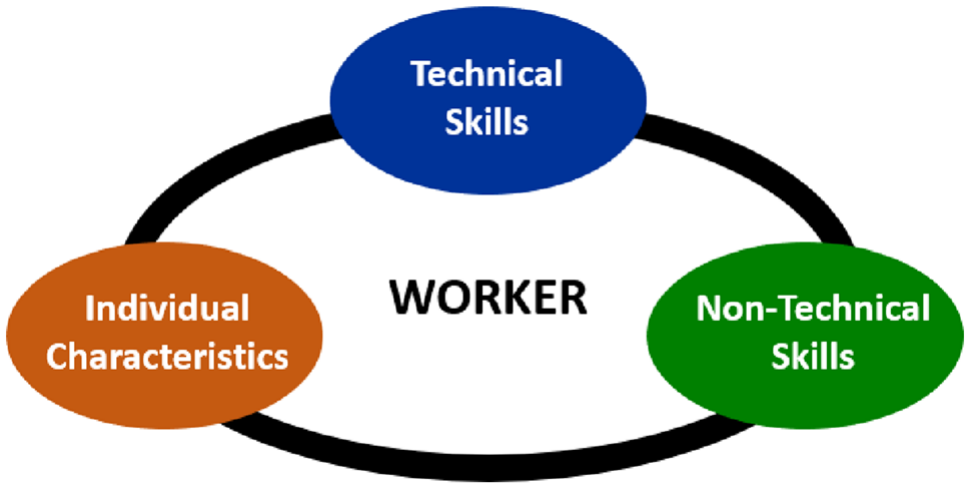
Non-technical skills, individual characteristics, and technical skills integration. Source: Authors (2020)

Analysing all these data, it is possible to notice that, in the workplace, non-technical skills do not include the technical skills required to get the job done, e.g., the know-how to operate a machine or execute a certain valve operation. However, NTS complement these technical skills and together with the individual characteristics of the workers, form their behaviour and performance to execute the tasks, efficiently and effectively. There is, therefore, an intrinsic integration between non-technical skills, individual characteristics, and technical skills, which is, ultimately, responsible for the behaviour and performance in the workplace. This intrinsic integration has a neurobiological reflex in the also intrinsically aggregated processing of rational and emotional data, characterizing and qualifying human social interactions (Lieberman, 2013). Figure 4 features a graphic representation of this intrinsic merge between non-technical skills, individual characteristics, and technical skills integration, responsible for the behaviour and performance of the worker.

In this sense, the natural human variabilities, contextualized in certain domain, is indeed a set of skills—technical and non-technical—and individual characteristics that are integrated and responsible for building the everyday resilience, both individual as much as collective. These variabilities are precisely what make the adjustment between the agility and urgency of work demands with the protocols, procedures and restrictions, allowing a dynamic balance between efficiency and thoroughness, particular in complex sociotechnical systems that form most of the workplaces in offshore and onshore process plants (Hollnagel, 2009). Examples of this dynamic variability promoting productivity and safety can be seen in both drilling and production operations onboard offshore platforms (França et al., 2022). Promoting this balance, which ultimately corresponds to the safety and productivity of the work performed, there is the workers individual abilities to response, based on their non-technical skills, individual characteristics and technical skills.

Analysing the responses from question “Considering only the graphic presentation and methodological structure, disregarding the questions themselves, which of the two sets of questions did you feel most comfortable answering?”, 22 of the 35 questionnaires, circa 63%, had selected the answer which has emoji and figure thumbnails, showing that there is a preference for visual characters. This result makes sense, once vision frequently dominates the human perception, when judging size, shape, position, perceiving the surroundings and all objects that form it (Ernst & Banks, 2002). This sense regulates balance and perception, working as a confirmation and integration of the other senses. Also, analysing the visual perception neurologically, a broad neural circuitry is used to process all the incoming stimulus, involving the amygdala and ventral striatum (Eagleman, 2015). These areas in the limbic system, which are among the most primitive parts of the brain, traditionally connects perception to action. This way, humans are essentially visual, perceiving, interacting and responding to external stimuli, translating sensory information from the external environment into meaningful internal representations that can be used to guide behaviour and assess risks (Carter et al., 2019). This human intrinsic neurobiological characteristic allows the situation awareness of the surroundings, as much as the risk perceptions of situations, places and interactions (Balcetis & Lassiter, 2010). Therefore, comparing written information with visual information, visual perception is the first step that precedes individual and collective answers for all external stimulus received, seeming reasonably to use this as an efficient way to promote safety.

## Conclusions

Analysing the set of competences needed to deal with unanticipated or critical events at the sharp end operations, such as FPSO or refineries operations, are the human variabilities responses at the system level, which form, ultimately, the system resilience. The system’s abilities to response are the set of the workers individual abilities to response, based on their non-technical skills, individual characteristics, and technical skills. And in the O&G industry, as can be seen by this research, this set is present in the sharp end of the operations of facilities/utilities, where workers, day by day, use all their skills to perform routine activities, control actions when there are near misses in the process, as well as acting in emergencies. In this sense, communication stands out as the most needed non-technical skills, in any situation, acting as a main line that bound together all the other skills and keep the workflow and development. The link between communication and teamwork is truly solid, reflecting, and at the same time being affected, by leadership and the organizational culture. And as much as this is solid, the decision-making process generates a significant increase in the assertiveness of decisions, both in the daily routine, as in critical situations that would precede accidents. However, some discoveries pointed out that situation awareness is still a non-technical skill that needs development, being highlighted by questionnaires and interviews as deficient in several situations. Bearing in mind that situational awareness is directly connected with individual and collective risk perceptions, there is a real need to develop it, since it is based on the analysis of what is happening in the sharp end, where actions are taken. Although this need of improvement, the results obtained indicated the existence of worker’s adaptability to the dynamic demands that they receive in the sharp end of the operations, counting not only with their non-technical skills, but also with all other competences. These competences are their technical and non-technical skills, as well as their individual characteristics, that linked together allow an adaptable performance for the dynamic requests of the work, both routinely and in emergencies. Indeed, this performance variabilities in the activities of utilities and facilities areas enables an integration between productivity and safety in the entire system, which ultimately characterizes its resilience. Therefore, these human adaptive variabilities resulting from their competences are not a problem of the system, but a solution response to the demands of the dynamic and complex functioning of the entire sociotechnical system.
